# Phylogenetic analysis of selected species of Asteraceae on the basis of *RPS* 11 Gene

**DOI:** 10.1038/s41598-024-75991-0

**Published:** 2024-10-22

**Authors:** Syeda Anber Zahra, Javed Iqbal, Banzeer Ahsan Abbasi, Sobia Kanwal, Mona S. Alwahibi, Mohamed S. Elshikh, Muhammad Rizwan, Rashid Iqbal, Tariq Mahmood

**Affiliations:** 1 School of Food and Pharmacy, Shanghai Zhongqiao Vocational and Technical University, Shanghai, 200000 China; 2 Department of Botany, Bacha Khan University, Charsadda, 24420 Khyber Pakhtunkhwa Pakistan; 3 Department of Botany, Rawalpindi Women University, 6th Road, Satellite Town, Rawalpindi, 46300 Pakistan; 4 Department of Biology and Environmental Sciences, Allama Iqbal Open University, Islamabad, 45320 Pakistan; 5 Department of Botany and Microbiology, College of Science, King Saud University, Riyadh, 11451 Saudi Arabia; 6 Institute of Crop Science and Resource Conservation (INRES), University of Bonn, 53115 Bonn, Germany; 7 Department of Agronomy, Faculty of Agriculture and Environment, The Islamia University of Bahawalpur, Bahawalpur, 63100 Pakistan; 8 Department of Life Sciences, Western Caspian University, Baku, Azerbaijan; 9 Department of Plant Sciences, Faculty of Biological Sciences, Quaid-i-Azam University, Islamabad, 45320 Pakistan

**Keywords:** *Asteraceae*, Genetic variability, *rps* 11, Phylogram, 3D protein modeling, Molecular evolutionary genetic analysis, Genetics, Plant sciences

## Abstract

The Asteraceae family is a prominent group of flowering plants found across the globe, with the exception of Antarctica. The Asteraceae family is a largest flowering family pivotal group in plant evolution and diversification. Despite its importance, the genetic diversity within this family remains understudied. We focused on the *rps*-11 gene, a chloroplast marker previously utilized in phylogenetic studies, to investigate its potential in resolving Asteraceae relationships. The focus was on examining genetic diversity within sixteen specifically chosen species from the Asteraceae family. This assessment was based on an analysis of a chloroplast gene responsible for encoding the ribosomal protein of the smaller subunit 11 (*rps* 11). Nearly 417 bp of *rps* 11 gene was amplified, sequenced, computationally translated into amino acid sequence and the data was used for phylogenetic analysis as well as for *rps* 11 protein structure predictions. Based on nucleotide and amino acid sequences phylograms were drawn with the help of Molecular Evolutionary Genetic Analysis (MEGA 6), which exhibited clear genetic relationship among species under investigation. The observed genetic distance was 0.02 for Maximum likelihood tree based on nucleotide sequences whereas it was 0.05 for phylogram based on amino acid sequences. These values revealed that amino acid-based tree has demonstrated greater diversity among selected species in comparison to nucleotides-based tree. On the basis of pair wise distance calculations, genetic divergence values were found within the range of 0.015–0.309. Moreover, 3D protein modeling for *rps* 11 protein of sixteen selected species was also carried out by iterative threading assembly refinement (I-Tasser) software. The models exhibiting the highest C-score were picked with satisfactory plot statistics (> 90%) and structurally validated by PROCHECK. Furthermore, Ramachandran plots displayed that the *rps* 11 protein structures of *Tagetes minuta*,* Xanthium strumarium*,* Lactuca sativa* and *Chrysanthemum indicum* have best feature models with > 90% of residues in the allowed region and ≤ 2% in the disallowed region. The research is not enough to stand alone to validate the viability of the *rps*11 gene as a prospective contender for phylogenetic analysis. İn future we will focus on the maximum genetic diversity theory for phylogenetic analysis of this family.

## Introduction

The family *Asteraceae* is recognized as the sunflower, aster or daisy family. It is the largest flowering plant family and about 10% of Angiosperm plants belong to this family^1,2^. According to the Royal Botanical Gardens of Kew, the family has around 1600 genera and 23,000 species^3^. *Asteraceae* is found worldwide except for Antarctica and has notable diversity, particularly in the subtropical and tropical regions of Southern Africa, the Mediterranean region, the Andes, North America, Eastern Brazil, Southwestern China and Central Asia^4,5^. Most distinguishing feature of this family is the arrangement of florets on a receptacle in centripetally emerging heads enclosed by bracts and anthers bonded in a sphere. Plants belonging to this family are mostly herbaceous^2^. Members of family *Asteraceae* are mostly perennial or biennial, annual herbs and rarely trees, sometimes bushes, with or without milky latex, sometimes with spines (Flora of Pakistan). Some of the genera of *Asteraceae* family *Aster*, *Helianthus*, *Chrysanthemum* and *Tagetes* etc. are ornamental plants and most of them have therapeutic values. The commercial importance of these plants can be assessed by the uses such as vegetable and food, as hair tonic etc.^6,7^

Chloroplast DNA (cpDNA) is the most preferred DNA molecule for phylogenetic analysis. cpDNA offer an exceptional opportunity for studying phylogeny at intra and interspecies level due to its extraordinary genetic potential and conserve nature as compared to nuclear DNA^8–11^. Several cpDNA genes (*mat*K, *rbc*L, *atp*B, *trn*L, *trn*L-F, *rps*11, *rps*14, *rps*16 and *ndh*F) have been used for understanding phylogeny in plants at different systematic levels in plant kingdom^4,5,8–16^. For assessment of taxonomic status and investigation of the evolutionary relationship among selected genera of family *Asteraceae* molecular marker based on chloroplast DNA was used^17,18^. In the present study, molecular and bioinformatics analyses were carried out to evaluate genetic variability among different species of family *Asteraceae* on the basis of a chloroplast gene *rps* 11.

## Materials and methods

### Collection of plant material

Selected plants of the *Asteraceae* family were collected from different geographical areas of Pakistan including Islamabad, Gujar Khan and Peshawar following established protocol and permission was obtained. Our plant study complies with relevant institutional, national and international guidelines and legislation. Further, the plant material was identified by Javed Iqbal and taxonomically validated by well-known taxonomist Dr. Syed Afzal Shah, Assistant Professor at Department of Biological Sciences, National University of Medical Sciences Rawalpindi, Pakistan. The voucher specimens were submitted to gene bank of the National University of Medical Sciences Rawalpindi, Department of Biological Sciences, for allotment of voucher number SAS-675, multiplication and to make sure availability for future use. (Table 1).

### DNA isolation and primer designing

Complete genomic DNA from fresh leaves was extracted using the CTAB (Cetyl Trimethyl Ammonium Bromide) method as demonstrated by Richards^19,20^. Forward and reverse primer of *rps*11 gene were designed by online web server primer 3 (version 4.0) (http//primer3. sourceforage.net/). The Tobacco cpDNA sequence (Accession # Z00044.2) available in Genbank was used to design *rps*11 gene primers. The sequence of forward and reverse primers is;

*rps* 11 Forward: 5’ TGGCAAAAGCTATACCGAAAA 3’.

*rps* 11 Reverse: 5’ TTCGGAGGTCTACAGCCATT 3’.

### Amplification and sequencing of rps 11 gene from different species of *Asteraceae*

To amplify *rps* 11 gene, 25 µL of reaction mixture containing 1 µl template, 1 µl (25 pmol) of each primer, 12.5 µl PCR Master Mix (Fermentas) and 9.5 µl nano pure water was prepared. The amplification conditions were 35 cycles of denaturation at 94 °C for 1 min, annealing at 55 °C for 30 s, and extension at 72 °C for 1 min, followed by a single-step of final extension at 72 °C for 20 min. The PCR products were confirmed on 1.5% agarose gel. Confirmed PCR products were purified with the help of the JETquick (Genomed) PCR Product Purification Kit and samples were sent to Macrogen (South Korea) for sequencing.

### Phylogenetic and protein structural analysis

Phylogenetic trees were constructed based on nucleotide as well as amino acid sequences of *rps* 11 representing sixteen selected species of *Asteraceae* to study the phylogenetic relationship by using Maximum Likelihood (ML). For 3D structural analysis, protein models were built using i-TASSER and were evaluated by Ramachandran plot analysis and PROCHECK. Ramachandran plot analysis and PROCHECK was used for protein structure validation. 3D models pdb files were uploaded on SAVES (The Structure Analysis and Verification Server) for Ramachandran plot and PROCHECK analysis.

## Results and discussion

**Fig. 1:**
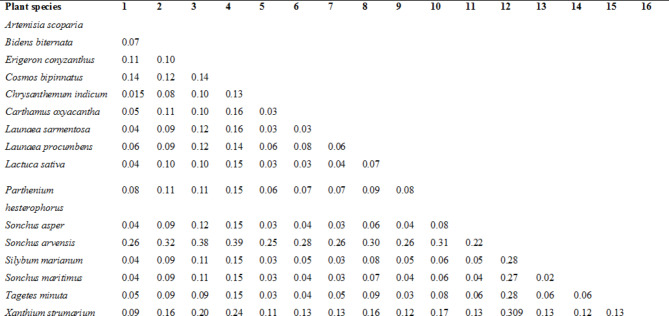
Estimates of evolutionary deviation for *rps* 11 sequences from sixteen plant species, calculated by pairwise distance using MEGA6.

Isolated genomic DNA of good quality from sixteen selected species of *Asteraceae* was run on 1% agarose gel for confirmation. After PCR, amplified products were loaded on 1.5% agarose gel and amplified product of ~ 417 bp was observed on the gel. All the sixteen amplified products representing sixteen species of *Asteraceae* were processed for sequencing and the final sequenced data was submitted to GenBank for the allotment of accession numbers as shown in the Table 1. Both nucleotide and amino acid sequences of *rps* 11 were used for inferring genetic characterization. Estimates of genetic variability among *rps* 11 sequences from sixteen plant species were calculated by pairwise distance using MEGA6 and moderate to low genetic diversity was observed which was in the range 0.015–0.309 (Fig. 1). The study does not provide sufficient evidence to support the *rps* 11 gene’s potential for use in phylogenetic analysis. Moreover, methodologies we used is based on neutral theory which is still debatable and uncertain. The validity of our findings is therefore contingent upon the limitations of this theoretical framework^21,22^. We recognize that alternative theories, such as the maximum genetic diversity theory offer a more comprehensive understanding of molecular evolution by incorporating the role of physiological selection and organismal complexity^23,]^ These alternative approaches may provide a more nuanced explanation of our results, and we encourage future studies to explore these perspectives^24,25^. The greatest genetic diversity idea will be our primary emphasis in future phylogenetic analyses of this family.

**Table 1:** Area of collection and GenBank accession numbers of chloroplast *rps* 11 gene of selected species of *Asteraceae.*

Serial no.	Plant Species	Location	Accession no.
1	*Artemisia scoparia*	Islamabad	KU859955
2	*Erigeron conyzanthus*	Islamabad	KU859957
3	*Cosmos bipinnatus*	Islamabad	KU859958
4	*Chrysanthemum indicum*	Islamabad	KU859959
5	*Carthamus oxyacantha*	Islamabad	KU859960
6	*Bidens biternata*	Peshawar	KU859956
7	*Launaea sarmentosa*	Islamabad	KU862618
8	*Launaea procumbens*	Islamabad	KU862619
9	*Lactuca sativa*	Islamabad	KU862620
10	*Parthenium hysterophorus*	Islamabad	KU862621
11	*Sonchus asper*	Islamabad	KU862622
12	*Sonchus arvensis*	Islamabad	KU862623
13	*Silybum marianum*	Islamabad	KU862624
14	*Sonchus maritimus*	Islamabad	KU862625
15	*Tagetes minuta*	Gujar khan	KU862626
16	*Xanthium strumarium*	Islamabad	KU726095

**Fig. 2:**
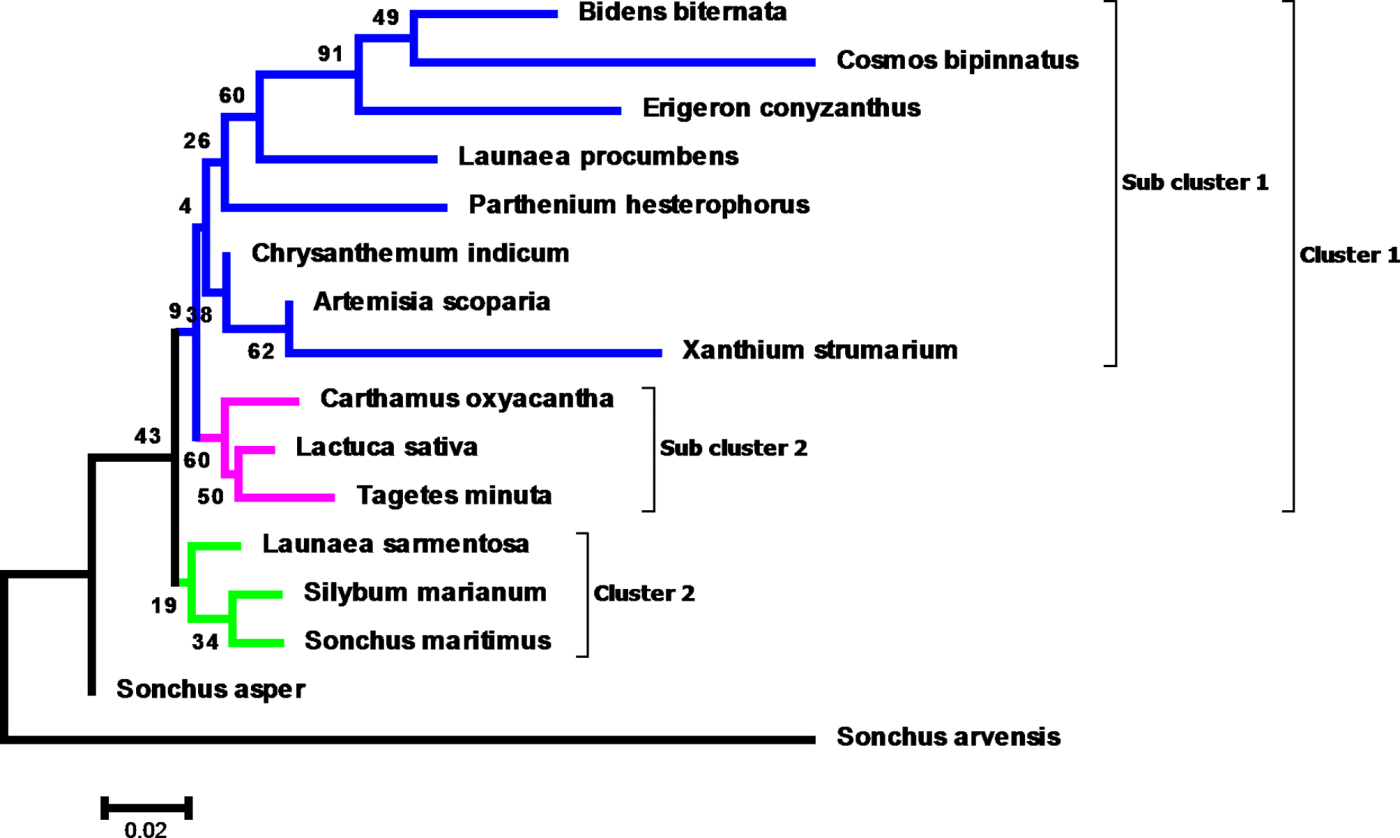
Phylogenetic tree based on *rps* 11 gene sequences demonstrating genetic variability among sixteen selected species of family *Asteracaea* using MEGA6.

A phylogram was prepared for the *rps* 11 gene sequences to establish the evolutionary relationship of sixteen species of *Asteraceae*. The phylogram displayed two major clusters, cluster I and cluster II (Fig. 2). Cluster I is comprised of eleven species namely *Artemisia scoparia*, *Bidens biternata*, *Erigeron conyzanthus*, *Cosmos bipinnatus*, *Chrysanthemum indicum*, *Carthamus oxyacantha*, *Launaea procumbens*, *Xanthium strumarium*, *Tagetes minuta*,* Parthenium hysterophorus and Lactuca sativa*. In this cluster bootstrap value was observed in the range of 4–91% which clearly indicated the phylogenetic relationship amongst the selected species. Highest bootstrap value 91% which shows highest similarity was found in *Erigeron conyzanthus*, *Bidens biternata* and *Cosmos bipinnatus* indicating common origin. On the basis of genetic divergence, this cluster was further divided into two subclusters, subcluster I and subcluster II. However, cluster II comprises of three species namely *Silybum marianum*, *Sonchus maritimus* and *Launaea sarmentosa*. *Sonchus asper* and *Sonchus arvensis* appeared as outgroups in cluster II.

**Fig. 3:**
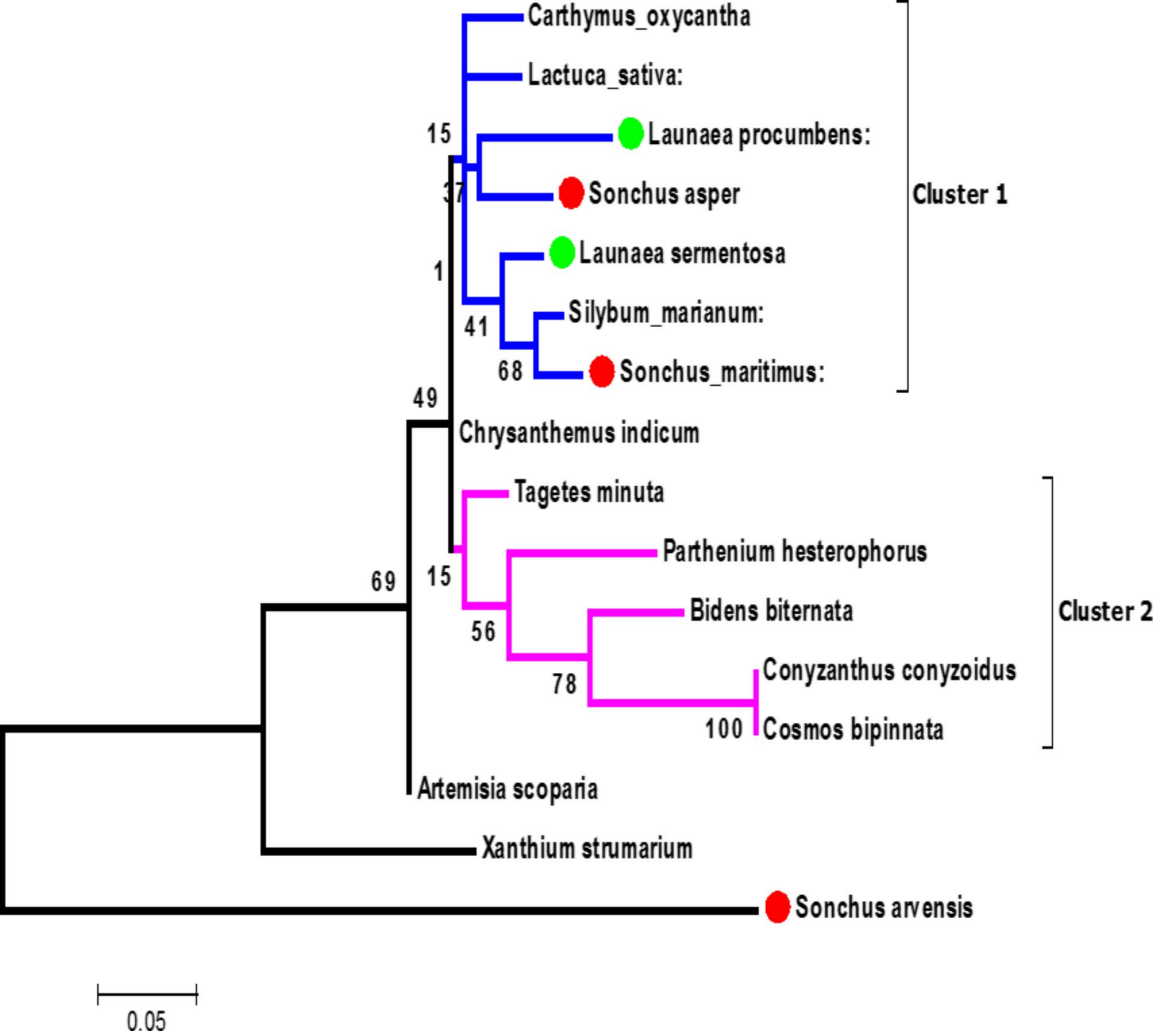
Phylogram representing genetic variability among sixteen selected species of family *Asteracaea* using MEGA6 based on *rps* 11 protein sequences.

The nucleotide data was translated into amino acid sequences using online tool TRANSLATE and translated amino acid sequence data was also used for inferring genetic characterization. A phylogenetic tree was constructed using MEGA 6, like the same was it was done for nucleotides data. Two major clusters represented by cluster I and cluster II (Fig. 3) were observed in this tree. In cluster I, there were seven species namely, *Carthamus oxyacantha*, *Launaea procumbens*, *Lactuca sativa*, *Silybum marianum*, *Sonchus maritimus*, *Sonchus asper* and *Launaea sarmentosa*. Highest bootstrap value 68 indicating maximum similarity was found in *Silybum marianum* and *Sonchus maritimus*, indicating their common origin.

Comparison of Maximum likelihood trees based on *rps* 11 nucleotide and amino acid sequences showed that protein-based tree has demonstrated higher diversity among studied species in comparison to nucleotide-based tree. The genetic distance was 0.02 in Maximum likelihood tree based on nucleotide sequences whereas it was 0.05 for amino acid base tree.

The bootstrap value range of 4–91% was seen for nucleotides based phylogenetic tree whereas it was 1-100% for phylogenetic tree based on amino acid sequences. Moreover, it was observed that *Sonchus arvensis* is placed as basal group in both phylograms demonstrating that it is an ancestor of cluster II. Our results support the findings of Amar et al. (2016) reporting higher genetic diversity among different species of *Asteraceae*^4^.

### Analysis of 3-dimensional protein structures

**Fig. 4:**
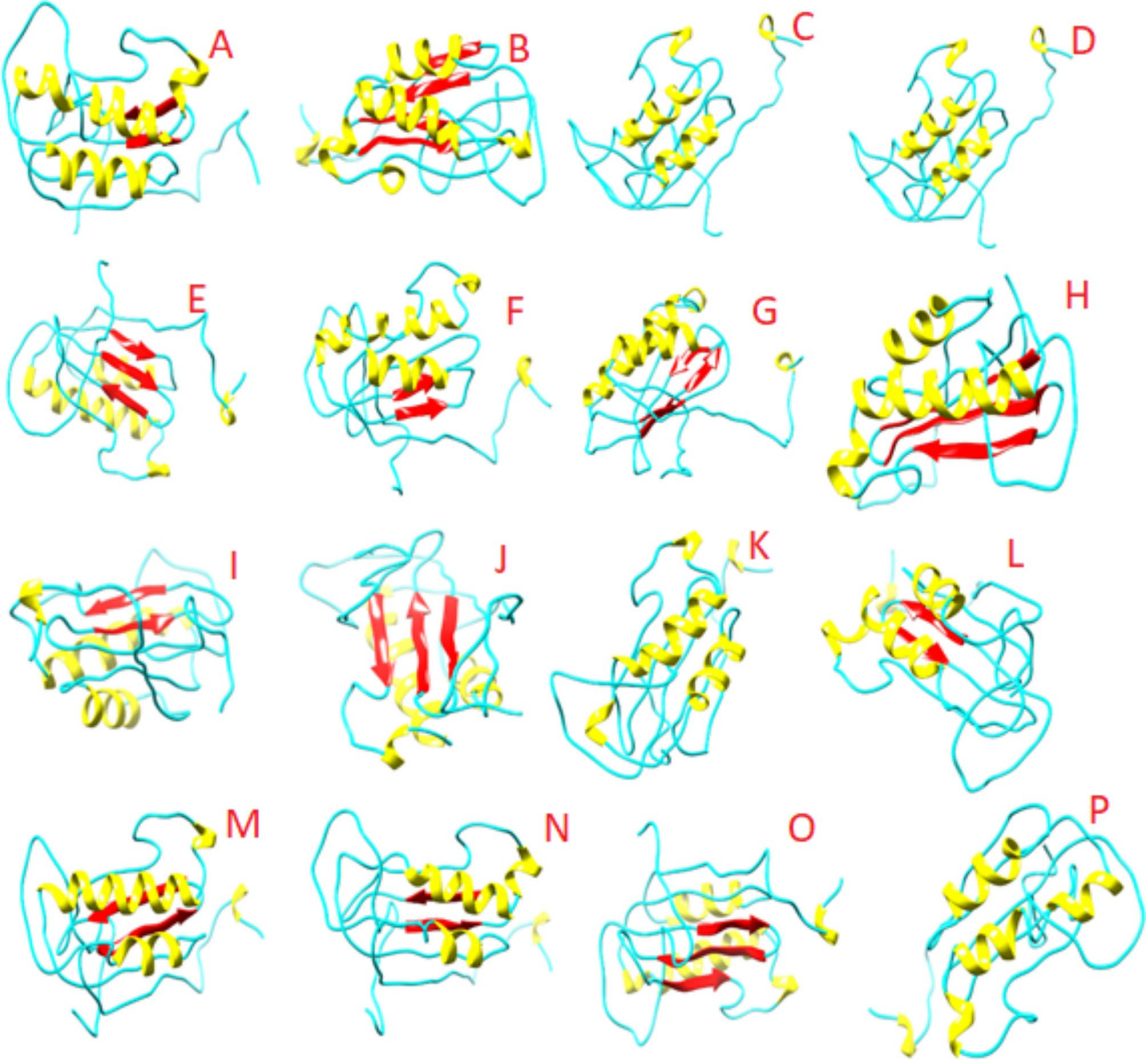
The 3D protein model with the highest C-score built by I-TASSER for different species of family Asteraceae A: *Artemisia scoparia*, B: *Bidens biternata*, C: *Erigeron conyzanthus*, D: *Cosmos bipinnatus*, E: *Chrysanthemum indicum*, F: *Carthamus oxyacantha*, G: *Launaea sermentosa*, H: *Launaea procumbens*, I: *Lactuca sativa*, J: *Parthenium hesterophorus*, K: *Sonchus asper*, L: *Sonchus arvensis*, M: *Silybum marianum*, N: *Sonchus maritimus*, O: *Tagetes minuta*, P: *Xanthium strumarium*

Three-dimensional Protein models were obtained using I-Tasser. I-Tasser produces five 3-dimensional models for each sequence alongside the confidence value. From 5 built structures for individually *rps* 11 protein, topmost model was chosen on the basis of higher percentages of residues in maximum allowed areas and minimum percentage scores in disallowed areas. The outcome suggested that model 1 for all sixteen species predicted by I-TASSER was good model and should be analyzed further. Model 1 of *rps* 11 from all sixteen species of *Asteraceae* was visualized using UCSF Chimera as shown in Fig. 4.

### Protein structure analysis and validation

**Fig. 5:**
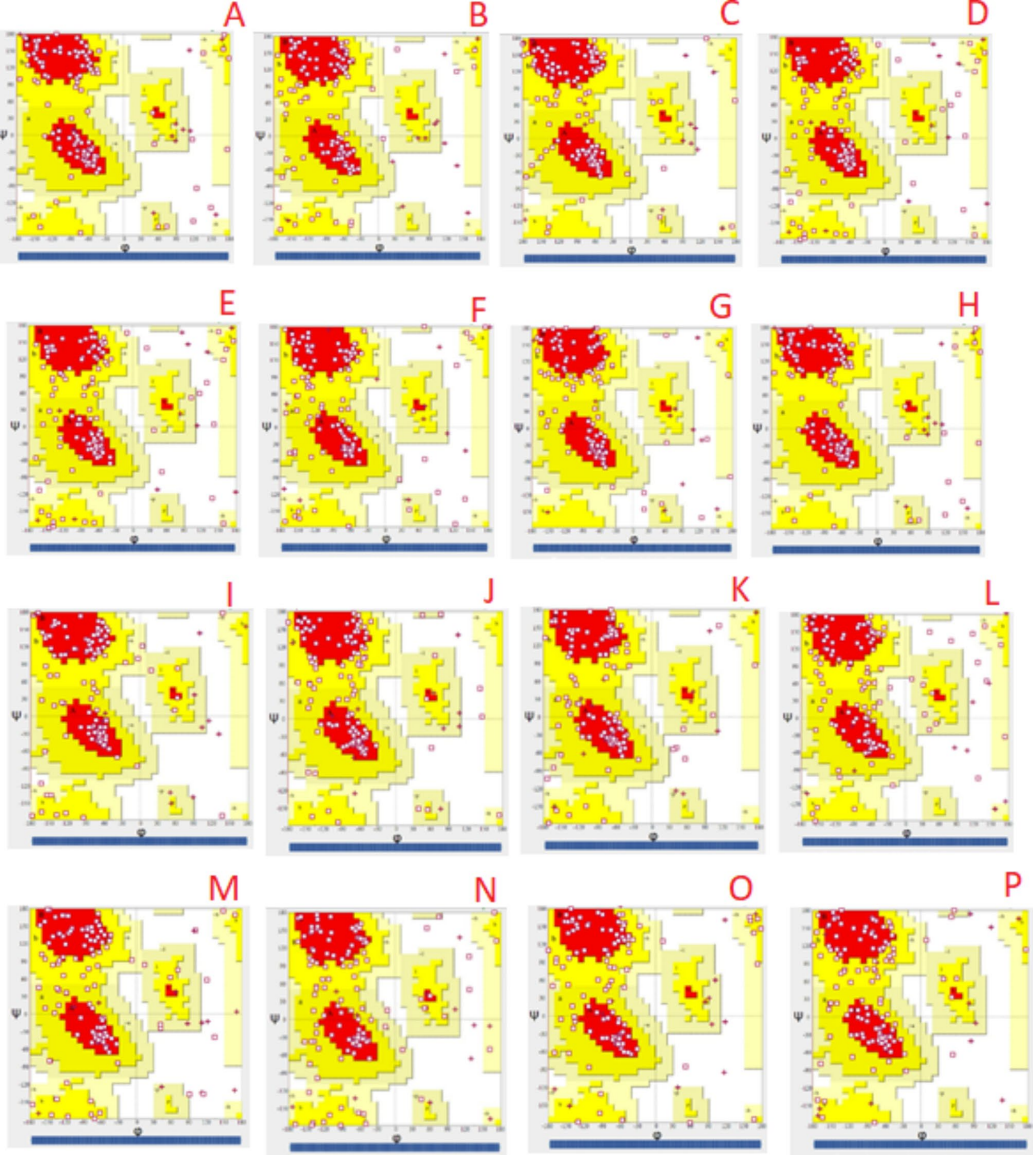
The Ramachandran plot for *rps* 11 protein of different species of family *Asteraceae* built by SAVES A: *Artemisia scoparia*, B: *Bidens biternata*, C: *Erigeron conyzanthus*, D: *Cosmos bipinnatus*, E: *Chrysanthemum indicum*, F: *Carthamus oxyacantha*. G: *Launaea sermentosa*, H: *Launaea procumbens*, I: *Lactuca sativa*, J: *Parthenium hesterophorus*, K: *Sonchus asper*, L: *Sonchus arvensis*, M: *Silybum marianum*, N: *Sonchus maritimus*, O: *Tagetes minuta*, P: *Xanthium strumarium*.

PROCHECK can be sued to construct Ramachandran plots of 3D protein models and exhibit the placement of amino acids in the “disallowed region” or “allowed region”. Best quality model exhibit about ≤ 2% residues in the disallowed region and ≥ 90% residues in the favored region of the plot. Estimation and verification consequences from Ramachandran plot assessment displayed that the *rps* 11 protein structures of *Tagetes minuta*,* Xanthium strumarium*,* Lactuca sativa* and *Chrysanthemum indicum* have best feature models with about > 90% of residues in the allowed region and ≤ 2% in the disallowed region (Table 2). Keeping in mind the Ramachandran scores it was observed that Ramachandran plot for all the sixteen sequences were considered satisfactory (Fig. 5). In an earlier study, Ramachandran plot study was carried out for the structural investigation of wound-inducible proteinase inhibitor-II gene^17^. Similarly in silico analyses were conducted for structural modeling of antioxidant proteins of spinach by Ramachandran plot analysis^18^. To the best of our knowledge this is the first report describing the 3D protein structure and Ramachandran plot analysis for *rps* 11 protein in *Asteraceae* family.

**Table 2:** Ramachandran scores of models for all the sixteen sequences predicted by PROCHECK.

S.N	Plant species	Allowed region	Disallowed region
1	*Artemisia scoparia*	93%	7.0%
2	*Bidens biternata*	94.8%	5.2%
3	*Carthamus oxyacantha*	97.4%	2.6%
4	*Chrysanthemum indicum*	98.2%	1.8%
5	*Erigeron conyzanthus*	95.7%	4.3%
6	*Cosmos bipinnatus*	95.7%	4.3%
7	*Lactuca sativa*	98.2%	1.8%
8	*Launaea procumbens*	96.5%	3.5%
9	*Launaea sermentosa*	94.7%	5.3%
10	*Parthenium hesterophorus*	97.4%	2.6%
11	*Silybum marianum*	95.6%	4.4%
12	*Sonchus arvensis*	93.6%	6.4%
13	*Sonchus asper*	95.7%	4.3%
14	*Sonchus maritimus*	97.4%	2.6%
15	*Tagetes minuta*	98.3%	1.7%
16	*Xanthium strumarium*	98.2%	1.8%

## Conclusion

Our study demonstrates the utility of the *rps*11 primer for phylogenetic analysis in the Asteraceae family. The genetic diversity revealed among the sixteen selected species highlights the complexity and richness of this family. The generated 3D protein models of the *rps*11 protein showed high structural quality, with satisfactory plot statistics and Ramachandran plots indicating accurate predictions. Notably, the models for Tagetes minuta, Xanthium strumarium, Lactuca sativa, and Chrysanthemum indicum exhibited exceptional quality, with over 90% of residues in the allowed region and less than 2% in the disallowed region. Furthermore, the 3D protein modeling of the *rps*11 protein offers a new perspective on the structural conservation and variation within this family. While our current research provides valuable insights, it is insufficient to independently confirm the *rps*11 gene’s potential as a reliable marker for phylogenetic analysis. In future studies, we plan to explore the application of the maximum genetic diversity theory to phylogenetic analysis of this family, which may offer a more comprehensive understanding of its evolutionary relationships.

## Data Availability

All the raw data in this research can be obtained from the corresponding authors upon reasonable request.

## **Declarations**

### Statement on guidelines

All experimental studies and experimental materials involved in this research are in full compliance with relevant institutional, national and international guidelines and legislation posing a conflict or bias.
